# Effect of Renal Sympathetic Denervation on Atrial Substrate Remodeling in Ambulatory Canines with Prolonged Atrial Pacing

**DOI:** 10.1371/journal.pone.0064611

**Published:** 2013-05-27

**Authors:** Xule Wang, Qingyan Zhao, He Huang, Yanhong Tang, Jinping Xiao, Zixuan Dai, Shengbo Yu, Congxin Huang

**Affiliations:** 1 Department of Cardiology, Renmin Hospital of Wuhan University, Wuhan City, People’s Republic (PR.) of China; 2 Cardiovascular Research Institute of Wuhan University, Wuhan City, PR. of China; The University of Manchester, United Kingdom

## Abstract

We have previously demonstrated that catheter-based renal sympathetic denervation (RSD) could suppress atrial fibrillation (AF) in canines with short-time rapid right atrial pacing (RAP). However, the role of renal denervation on atrial remodeling is unclear. The aim of the present study was to explore the long-term effect of RSD on the atrial remodeling during prolonged RAP. Twenty mongrel dogs were implanted with a high-frequency cardiac pacemaker with a transvenous lead inserted into the right atrial appendage. The dogs were divided into three groups: a sham-operated group (n = 6), the chronic RAP (CRAP) group (n = 7), and the CRAP+RSD group (n = 7). In the CRAP+RSD group, a pacemaker was implanted 6 weeks after RSD was performed bilaterally for recovery. RAP was maintained for 5 weeks in CRAP group and CRAP+RSD group. The plasma levels of Angiotensin II and aldosterone were significantly increased in CRAP group compared with sham-operated group, but the increasing trend was inhibited in CRAP+RSD group compared with CRAP group (P<0.05). Similarly, RSD suppressed the increasing trend that prolonged RAP produced in the left atrial levels of ANP, TNF-α and IL-6. Compared with the sham-operated group, the CRAP group had significantly increased levels of caspase-3, bax and Cx40 whereas the level of Bcl-2 decreased (P<0.05). RSD markedly reduced the upregulation of caspase-3, bax and Cx40 and the downregulation of Bcl-2 expression compared with the CRAP group (P<0.05). Picric acid–sirius red staining study suggested that RSD could markedly alleviate the lesion degree of cardic fibrosis induced by CRAP (P<0.05). Immunohistochemistry results showed that the densities of TH- and GAP43- positive nerves were significantly elevated in the CRAP group compared with the sham-operated group, while RSD operation signicantly inhibited the these changes produced by CRAP. These findings suggest that renal denervation could suppress the atrial remodeling after prolonged RAP in ambulatory canines.

## Introduction

Studies have indicated that the renin-angiotensin-aldosterone system (RAAS) has been shown to an important hormonal system in the initiation and pathogenesis of atrial fibrillation (AF) [1], [2], [3]. Inhibitors of the RAAS, such as angiotensin converting enzyme inhibitors and angiotensin receptor blockers, are now emerged as novel drugs for the prevention and treatment of AF [4], [5], [6]. Although these drugs may not possess direct anti-arrhythmic properties, a large number of research on AF have shown that RAAS blockade has beneficial effects on cardiac remodeling, which is specally related to RAAS inhibition [7], [8], [9], [10].

The important mechanism of AF known as atrial remodeling, which has been shown in previous studies, takes the form of atrial fibrosis, inflammation, apoptosis, gap junctional remodeling and neural remodeling [11], [12], [13], [14], [15]. Several studies have shown that hyperactivity of RAAS has been implicated in the cardiac fibrosis, inflammation and apoptosis in cardiovascular disease, such as increased angiotensin II and aldosterone levels. Furthermore, inhibition of the renin-angiotensin system decreases cardiomyocyte fibrosis, apoptosis and inflammation [16], [17], [18], [19].

Catheter-based renal artery ablation selectively reduces both renal sympathetic efferent and afferent nerve activity and causes renal sympathetic denervation (RSD). Some clinical trials and animal experiments have suggested that RSD has achieved a breakthrough in the treatment of resistant hypertension, which might have related to the inhibition of chronic sympathetic nervous overactivation [20], [21], and further has great potential in the treatment of heart failure, left ventricular hypertrophy, refractory AF and post-myocardical infarction cardiac remodeling [22], [23], [24], [25]. In a recent report from our laboratory [26], we demonstrated that RSD could suppress AF in canines with 7 h rapid right atrial pacing (RAP) and decrease the activity of RAAS. However, whether renal denervation can attenuate atrial substrate remodeling induced by CRAP is unclear. The aim of the present study was to explore the long-term effect of RSD on the atrial remodeling during chronic RAP (CRAP).

## Methods

### Experimental Animals and Study Protocol

The study protocol was approved by the Ethical Committee of the Wuhan University School of Medicine, and all animal handling was performed in accordance with the Wuhan Directive for Animal Research and the current Guidelines for the Care and Use of Laboratory Animals published by the National Institutes of Health (NIH publication no. 85–23, revised 1996). Twenty adult mongrel dogs (weight, 17 to 23 Kg) were supplied by the center of experimental animal in medical college of Wuhan University, and all owners of the dogs agreed to have their animals involved and provided a statement of informed consent. All dogs in this study were assigned randomly to three groups. Sham-operated group consisted of 6 dogs that were implanted with pacemakers without pacing; CRAP group consisted of 7 dogs that were implanted with pacemakers with RAP for 5 weeks; and CRAP+RSD group consisted of 7 dogs that first underwent catheter-based RSD and then were implanted with pacemakers after they recovered from RSD.

All of the dogs were anaesthetized with pentobarbital sodium (30 mg⋅kg^−1^, i.v.), intubated and ventilated with room air supplemented with oxygen from a respirator (MAO01746, Harvard Apparatus Holliston, USA). Normal saline at 50 to 100 ml⋅h^−1^ was infused to replace spontaneous fluid losses. Standard surface 12-lead electrocardiograms (MAC1200, GE, USA) were monitored continuously throughout the procedure. Under fluoroscopy, an atrial passive-fixation bipolar steroid-eluting tined endocardial pacing electrode (1642T, St. Jude Medical, Inc., USA) was delivered to the right atrial appendage via the right external jugular vein and connected to a high-rate cardiac pacemaker (Fudan University, Shanghai, China), which was implanted in a subcutaneous pocket of the neck. The pacemaker was attached to the endocardial lead and immediately operated in AOO mode (450 beats per minute, 0.2 ms pulse width with voltages of 5V) for 5 weeks.

In the CRAP+RSD group dogs, a 6F ablation catheter (Biosense Webster, Inc., Diamond Bar, CA, 91765, USA) was inserted into each renal artery via the femoral artery under fluoroscopy. We applied discrete, radiofrequency ablations of 6 Watts or less and lasting up to 80 seconds (s) each within each renal artery. During ablation, the catheter was advanced and retreated three times. The catheter system monitored tip temperature and impedance and altered its radiofrequency energy delivery in response to a predetermined algorithm. Antibiotics were administered for 3 days after the ablation. The effectiveness and safety of the RSD operation was demonstrated in our previous study [26].

Furthermore, a hemostatic sheath was inserted into the left femoral artery, and femoral artery blood pressure (BP) was measured at the baseline, 6 weeks after RSD and 5 weeks after RAP in CRAP+RSD group. Similarly, BP was measured in sham-operated group and CRAP group at the baseline and at the endpoint of the protocol.

### Electrophysiological Measurement

Hemostatic sheaths were inserted into the right femoral veins of the dogs at the baseline state and again after 5 weeks’ pacemaker implantation in all groups. Multi-polar electrode catheters were introduced into the femoral vein and placed in the high right atrium (HRA). The atrial effective refractory period (AERP) was determined in the HRA with an atrial pacing cycle length of 250 ms. The S1–S2 intervals were decreased from 180 ms to refractoriness initially by decrements of 10 ms (S1:S2_8∶1). As the S1–S2 intervals approached the AERP, decrements were reduced to 2 ms. An S1S1 programmed stimulation method (120 ms three times for 5 s) was adopted to induce AF.

### ELISA

Two millilitres of venous blood were collected in Ethylene Diamine Tetraacetic Acid (EDTA) vacutainers and centrifuged at 3 000 gravities for 10 min at 4°C at the baseline and at the endpoint of the protocol. The plasma was separately kept in microtubes and stored at −80°C until assay. At the completion of the protocol, the animals were euthanized and the hearts were quickly excised. Tissue specimens were obtained from the left atria (LA) and were temporarily stored at −80°C until assay. The plasma levels of Angiotensin II (Ang II) and aldosterone and the tissue levels of Atrial Natriuretic Peptide (ANP), Tumor Necrosis Factor-α (TNF-α), Interleukin-6 (IL-6), were examined by Enzyme linked Immunosorbent Assay (ELISA).

### Western Blotting

Forty micrograms of total protein from homogenized atrial tissue were solubilized for 5 min at 95°C in one volume-loading buffer and loaded onto a 5% SDS-PAGE gel, then transferred to polyvinylidene fluoride (PVDF) membranes. The membranes were blocked with 5% nonfat dry milk in Tris-buffered saline with Tween (TBST) (containing 0.5% TBST) for 1 hour and incubated overnight at 4°C with the primary antibodies (monoclonal rabbit anti-caspase-3, anti-B-cell Lymphoma Gene 2 (Bcl-2), mouse anti-bax, anti-Connexin 43 (Cx43) antibodies; Abcam, Inc., UK; used at 1∶1000; goat anti-Connexin 40 (Cx40) antibody, Santa, Inc., USA; used at 1∶500; rabbit anti-actin antibody, Santa, Inc., USA; used at 1∶1000). They were then washed in TBST three times, incubated with horseradish peroxidase (HRP)-conjugated second antibody for 1 hour at 37°C, and revealed by Immun-Star HRP Substrate. The relative expression of protein was determined with image analyzer software (AlphaEase FC, USA).

### Picric Acid–Sirius Red Staining

All samples for histology were fixed in 4% paraformaldehyde fixative until embedded in paraffin. The paraffin-fixed left atrial specimens were sliced into 6-µm-thick sections. Then, the sections were mounted on glass slides and stained with picric acid–sirius red (0.1% sirius red in saturated aqueous picric acid) to detect cardiac fibrosis. Stained sections were examined with fluorescence microscope (NIKON Ti-s, Japan). Collagen fiber presents a red color, while myocardial muscle fiber appears yellow. Three randomly selected images per section were digitally captured (magnification ×400) and the volume fraction of collagen was analyzed using the Image-Pro Plus 6.0 image software (IPP 6.0, Media Cybemetics, Georgia, USA).

### TUNEL Staining

For further detection of apoptosis, TUNEL staining was performed using a commercial kit (in situ Cell Death Detection Kit, Roche Bio-chemicals, Mannheim, Germany). Stained sections were examined with fluorescence microscope (Nikon TE2000, Japan) at EX 450–500 nm/EM515–565 nm. The cells that exhibited condensed nuclei with an irregular form or split into brown particles were considered to be TUNEL-positive cells. Each slide was examined under a microscope with 40× objectives to select 3 fields with the optical density of cell death using the IPP 6.0 software.

### Immunohistochemistry

All samples for histology were fixed in 4% paraformaldehyde fixative until embedded in paraffin. Four-micrometer sections were cut from paraffin blocks of the LA and right atria (RA). The sections were stained with tyrosine hydroxylase (TH) (monoclonal rabbit anti-TH antibody, Abcam, Inc., UK; used at 1∶500) to label sympathetic nerves and growth-associated protein 43 (GAP43) (monoclonal rabbit anti-GAP43, Millipore, Inc., USA; used at 1∶200) for growing nerve cones. Furthermore, the sections were stained with Cx40 (monoclonal goat anti-Cx40, Santa, Inc., USA; used at 1∶500) to prove the gap junctional remodeling. We determined the density by a computer-assisted IPP 6.0 software. The slides were coded so that the investigator who counted the densities was blinded to the dog identification at the time of nerve count. Each slide was examined under a microscope with 40× objectives to select 3 fields with the highest density of nerves. The computer automatically detected the stained nerves in these fields by their brown color, and then calculated the nerve area occupied by the nerves in the field. The density was the positive area divided by the total area examined. The mean density in these 3 selected fields was used to represent the density of that slide.

### Statistical Analysis

Data are expressed as mean±SD. Two-sample independent student’s *t-*tests were used to compare the means of two groups. One-way analysis of variance (ANOVAs) with Neuman-Keuls tests were used to compare the means of continuous variables among multiple groups; cases of significant difference were further analyzed with the Tukey-Kramer test. All statistical tests were two-sided, and a probability value <0.05 was required for statistical significance.

Results of immunohistochemistry were compared between the three groups using a Kruskal Wallis test. In the event the p<0.05 the different groups were mutually compared using a Mann – Whitney test.

## Results

Of the 20 dogs, successful experiments were performed on 18 (body weight, 19±2 Kg; range, 17 to 23 Kg). One dog died suddenly 3 days after RAP, possibly because the atrial electrode moved into the ventricle. The other dog died 20 days after RAP because of an atrial punch that was confirmed by autopsy. A total of 18 dogs completed the study protocol a mean of 5 weeks after the pacemaker was implanted. The dogs in CRAP+RSD group required 6 weeks for recovery from RSD before they were implanted with pacemakers. The total duration of monitoring was 6±2 weeks for sham-operated group dogs, 5±1 weeks for CRAP group dogs, and 11±1 weeks for CRAP+RSD group dogs.

### Effect of RSD on Blood Pressure

Table 1 summarizes the average systolic BP and diastolic BP responses in all dogs. Systolic BP was significantly decreased after RSD for 6 weeks in CRAP+RSD group (P = 0.001). Similarly, systolic BP was decreased after RAP for a mean of 5 weeks in CRAP group (P = 0.001) and CRAP+RSD group (P = 0.021). However, compared with the CRAP group dogs, the CRAP+RSD group dogs had a lower SBP (P = 0.036) at the endpoint of the study.

**Table 1:** Changes in BP among the sham-operated, CRAP, and CRAP+RSD groups before and after rapid atrial pacing.

	systolic BP (mmHg)	diastolic BP (mmHg)
Sham-operated group		
Baseline	169±13	102±14
Endpoint	163±7	105±8
CRAP group		
Baseline	168±15	103±15
Endpoint	131±6** ††	86±9††
CRAP+RSD group		
Baseline	174±11	104±20
6 weeks after RSD	136±5**	93±10
Endpoint	108±14** †† ‡	73±17* †

BP, blood pressure.

* P<0.05,

** P<0.01 vs baseline;

† P<0.05,

†† P<0.01 vs sham-operated group;

‡ P<0.05 vs CRAP group.

### Effect of RSD on Electrophysiology Induced by CRAP

There were no significant differences in AERP or the inducibility of AF at the baseline state among three groups. At the endpoint of the study, compared the sham-operated group, AERP in the CRAP group was significantly decreased. While the AERP exhibited a decreasing trend compared the CRAP+RSD group with the sham-operated group, the probability value was not significantly different (p = 0.054). During S1S1 programmed stimulation (120 ms three times for 5 s), CRAP group dogs exhibited higher numbers of inducements compared to sham-operated group (2.3±0.6 vs 0.4±0.5, p = 0.012) and CRAP+RSD group dogs (2.3±0.6 vs 0.8±0.9, p = 0.034).

### Effect of RSD on the Plasma Levels of Ang II and Aldosterone Induced by CRAP

As Table 2 shows, compared with the baseline, CRAP group showed a statistically significant increase in the plasma levels of Ang II and aldosterone at the endpoint of the protocol (P<0.01); similarly, CRAP+RSD group dogs had a significant increase in the plasma levels of Ang II and aldosterone (P<0.05). There were no significant differences in the plasma levels of Ang II and aldosterone between the baseline and the endpoint of the study in the sham-operated group. At the endpoint of the protocol, compared with sham-operated group, CRAP group showed a statistically significant increase in the plasma levels of Ang II and aldosterone (P<0.05); rather, the plasma levels of Ang II and aldosterone were significantly reduced after RSD compared with the values for the CRAP group.

**Table 2:** Changes in the plasma levels of Ang II and aldosterone among the sham-operated, CRAP, and CRAP+RSD groups before and after rapid atrial pacing (ng/l).

	Ang II	aldosterone
Sham-operated group		
Baseline	80.5±13.1	258.4±49.8
Endpoint	105.0±25.2	297.4±74.2
CRAP group		
Baseline	76.8±16.0	288.6±52.8
Endpoint	217.5±53.5** ††	671.4±124.3** †
CRAP+RSD group		
Baseline	84.4±15.4	289.2±53.6
Endpoint	149.6±26.3** † ‡	400.7±61.6* ‡‡

Ang II, Angiotensin II.

* P<0.05,

** P<0.01 vs baseline;

† P<0.05,

†† P<0.01 vs sham-operated group;

‡ P<0.05,

‡‡ P<0.01 vs CRAP group.

### Effect of RSD on Atrial inflammation Induced by CRAP

Table 3 suggests the changes in the left atrial levels of ANP, TNF-α and IL-6. Compared with sham-operated group, CRAP group showed a statistically significant increase in left atrial levels of ANP, TNF-α and IL-6 (P<0.05); the levels of ANP and TNF-α in RAP+RSD group were significantly increased (P<0.05), while the increasing trend in the level of IL-6 was not statistically significant (P = 0.157). Compared with the CRAP group dogs, the CRAP+RSD group dogs had lower levels of ANP, TNF-α and IL-6 (P<0.05). Thus, RSD suppressed the increasing trend that prolonged RAP produced in the levels of ANP, TNF-α and IL-6.

**Table 3:** Changes in the left atrial levels of ANP, TNF-α and IL-6 among the sham-operated, CRAP, and CRAP+RSD groups after rapid atrial pacing (ng/l).

	ANP	TNF-α	IL-6
Sham-operated group	61.2±10.7	10.9±2.8	8.7±2.1
CRAP group	169.4±27.6**	83.4±10.5**	19.9±4.5**
CRAP+RSD group	108.7±16.0* †	45.6±5.7* ††	11.2±3.6†

ANP, Atrial Natriuretic Peptide; TNF-α, Tumor Necrosis Factor-α; IL-6, Interleukin-6.

* P<0.05,

** P<0.01 vs sham-operated group;

† P<0.05,

†† P<0.01 vs CRAP group.

### Effect of RSD on Atrial Fibrosis Induced by CRAP

**Figure 1:**
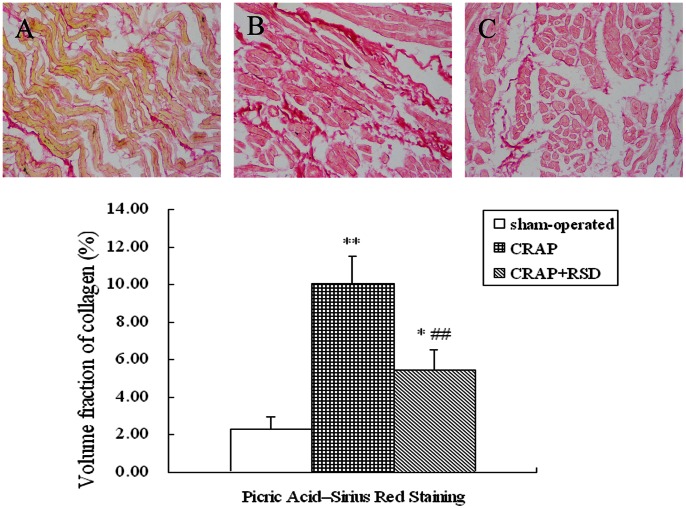
Effect of RSD on collagen fibers in the left atria detected by picric acid–sirius red staining (light microscope). A: sham-operated group; B: the CRAP group; C: the CRAP+RSD group. (original magnification: ×400). *P<0.05, **P<0.01 vs sham-operated group; ^##^P<0.01 vs CRAP group.

When stained by Picric acid–Sirius red and studied using fluorescence microscopy, the sections presented the different colors in the regions. The color and morphology always permit clear distinction from collagen and myocardial muscle fiber. As shown in Figure 1, staining for collagen showed a significantly increasing intercellular space in both the CRAP group (P = 0.000) and the CRAP+RSD group (P = 0.024) compared with the sham-operated dogs. In comparison, the RSD operation in the paced dogs clearly decreased the volume fraction of collagen compared with that of the CRAP group (P = 0.008).

### Effect of RSD on Cardiocyte Apoptosis Induced by CRAP in Canine Atria

**Figure 2:**
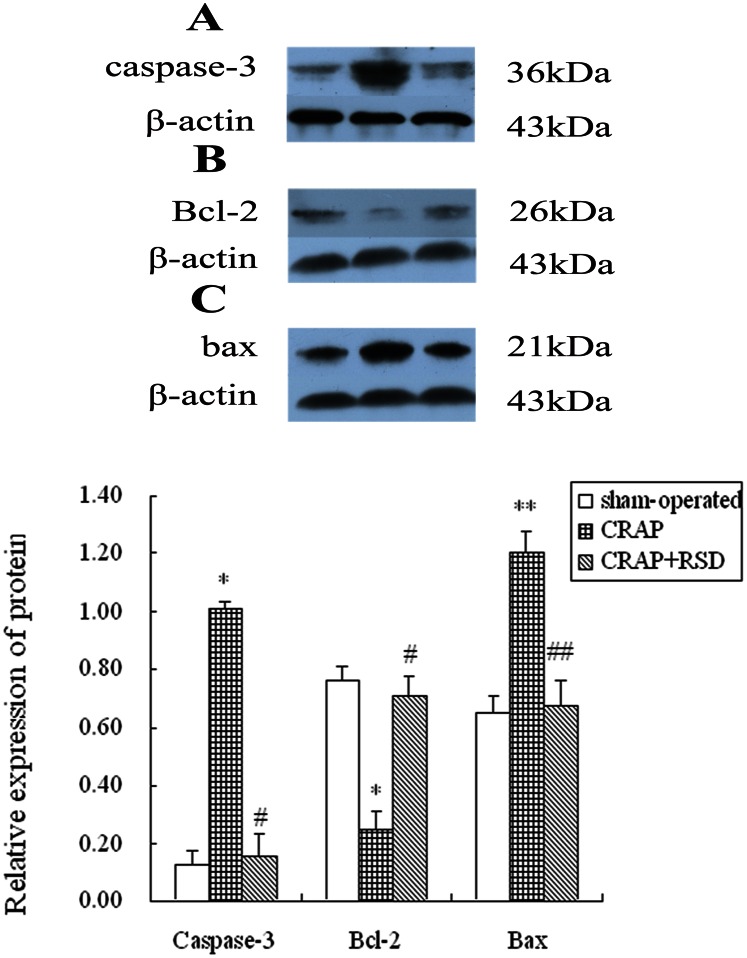
Representative Western blots of apoptosis-associated protein samples extracts from the left atrium. (A) Examples of the western blot bands recognized by the caspase-3 antibodies (36 kDa) for sham-operated, CRAP and CRAP+RSD groups. (B) Western blots for Bcl-2 with a specific band at 26 kDa were performed for sham-operated, CRAP and CRAP+RSD groups. (C) A specific band at 21 kDa for bax was detected in left atrial tissue of sham-operated, CRAP and CRAP+RSD groups. Antibodies for β-actin with a specific band at 43 kDa were used as reference. Bcl-2, B-cell Lymphoma Gene 2. *P<0.05, **P<0.01 vs sham-operated group; ^#^P<0.05, ^##^P<0.01 vs CRAP group.

The western blotting analysis of caspase-3, Bcl-2 and bax proteins is presented in Figure 2. Quantitative analysis showed that the CRAP group had significantly increased levels of caspase-3 (P = 0.012) and bax (P = 0.005) compared with the sham-operated group, whereas the level of Bcl-2 (P = 0.022) decreased. RSD markedly suppressed the upregulation of caspase-3 (P = 0.027) and bax (P = 0.007) and the downregulation of Bcl-2 expression (P = 0.037) compared with the CRAP group.

**Figure 3:**
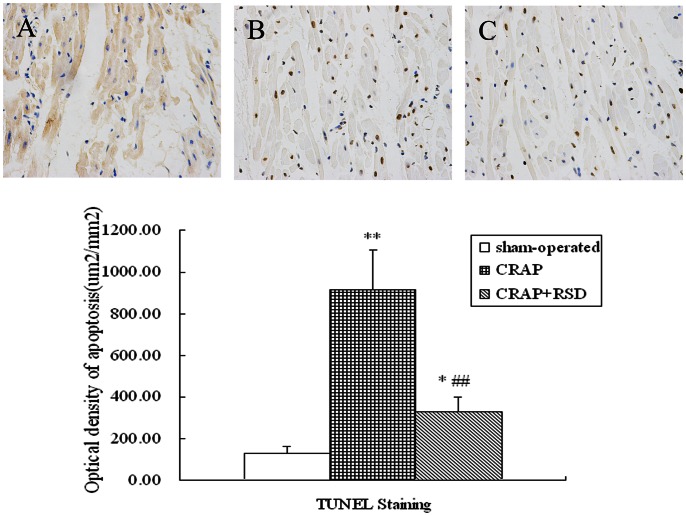
Effect of RSD on cardiocyte apoptosis in left atria detected with TUNEL staining. A: sham-operated group; B: the CRAP group; C: the CRAP+RSD group. (original magnification: ×400). *P<0.05, **P<0.01 vs sham-operated group; ^##^P<0.01 vs CRAP group.

The representative TUNEL staining was shown in Figure 3. Compared with sham-operated dogs, the optical density of TUNEL-positive cells was markedly increased in LA of the CRAP group after 5 weeks of atrial pacing (P = 0.000). Compared with CRAP group, RSD treatment markedly reduced cardiocyte apoptosis with significant difference (P = 0.003).

### Effects of RSD on Gap Junctional Remodeling Induced by CRAP in the Atria

**Figure 4:**
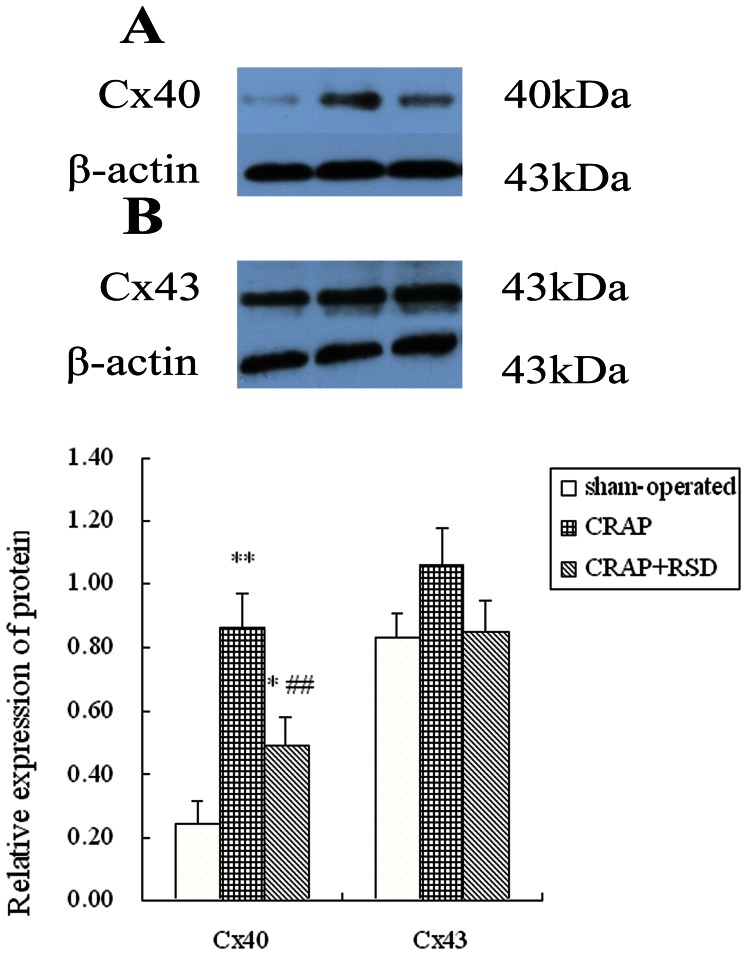
Cx40 and Cx43 protein expression detected with western blotting analysis. (A) A specific band at 40 kDa for Cx40 was detected in left atrial tissue of sham-operated, CRAP and CRAP+RSD groups. (B) Western blots for Cx43 with a specific band at 43 kDa were performed for sham-operated, CRAP and CRAP+RSD groups. Monoclonal antibodies for β-actin with a specific band at 43 kDa were used as reference. Cx40, Connexin 40; Cx43, Connexin 43. *P<0.05, **P<0.01 vs sham-operated group; ^##^P<0.01 vs CRAP group.

Figure 4 shows the western blotting analysis of Cx40 and Cx43 proteins. Quantitative analysis showed that Cx40 protein expression increased significantly in the CRAP group compared with the sham-operated group (P = 0.000). While RSD inhibited the upregulation of Cx40 that was induced by 5 weeks of atrial pacing (P = 0.000). However, the expression levels of Cx43 were not significantly different among three group (P = 0.153).

**Figure 5:**
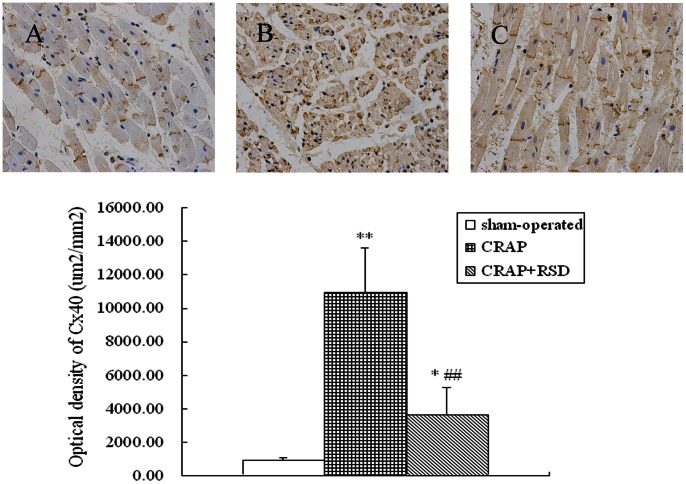
Immunostaining results of Cx40 expression in the atrium among all groups. A: sham-operated group; B: the CRAP group; C: the CRAP+RSD group. (original magnification: ×400). *P<0.05, **P<0.01 vs sham-operated group; ^##^P<0.01 vs CRAP group.

Figure 5 compares the immunostaining results of Cx40 expression in the atrium among all groups. Compared with sham-operated group, the Cx40-positive densities within the atria were significantly higher in both CRAP group and CRAP+RSD group (P<0.05). While CRAP+RSD group dogs had a lower density of Cx40 (P = 0.005) compared with the CRAP group.

### Effects of RSD on Neural Remodeling Induced by CRAP in the Atria

**Figure 6:**
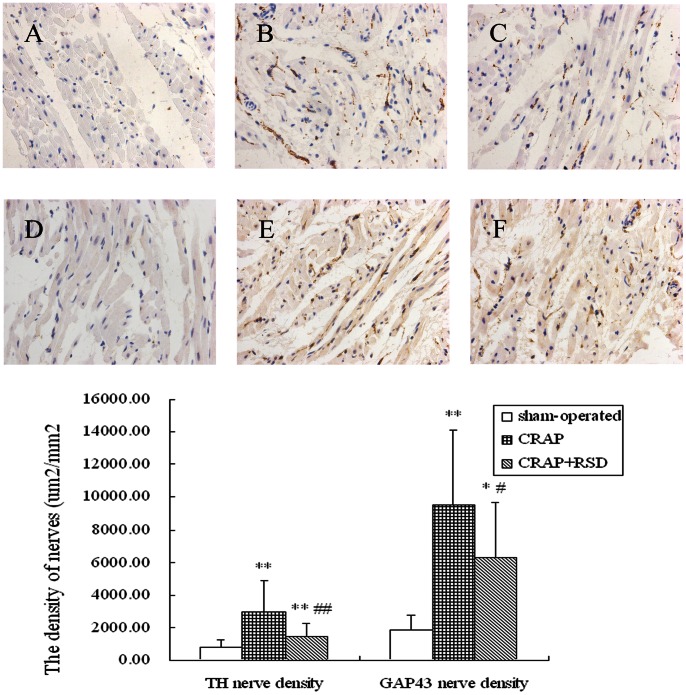
Histological sections of TH and GAP43 atrial nerves in all groups. Exemplary pictures of TH staining in the sham-operated group (A), the CRAP group (B) and the CRAP+RSD group (C); representative GAP43 staining of atrial myocardium in the sham-operated group (D), the CRAP group (E) and the CRAP+RSD group (F). (original magnification: ×400). TH, tyrosine hydroxylase; GAP, growth-associated protein. *P<0.05, **P<0.01 vs sham-operated group; ^#^P<0.05, ^##^P<0.01 vs CRAP group.

Figure 6 compares the immunostaining results of TH and GAP43 atrial nerves among all groups. The atrial nerve density for each group was expressed as a mean of nerve densities in the LA and RA. Compared with sham-operated group, the densities of TH-positive nerves within the atria were significantly higher in both CRAP group (P = 0.000) and CRAP+RSD group (P = 0.001). While CRAP+RSD group dogs had a lower density of TH-positive nerves (P = 0.003) compared with the CRAP group. Similarly, the GAP43-positive nerve densities were higher in both CRAP group (P = 0.000) and the CRAP+RSD group (P = 0.013), compared with the sham-operated group; RSD markedly suppressed the trend toward increased GAP43 nerve density (P = 0.035) compared with the CRAP group.

## Discussion

The principal purpose of this investigation was to assess the effects of RSD on atrial substrate remodeling during prolonged RAP. Results showed that RSD could suppress the AF vulnerability, the renin-angiotensin-aldosterone systematic activity and atrial remodeling, including the significantly increased atrial fibrosis, inflammation and apoptosis, together with atrial gap junctional remodeling and neural remodeling that are produced by long-term RAP in ambulatory canines.

### Chronic RAP, Catheter-based RSD and RAAS

In 1995, Morillo and colleagues [11] found that the canine model of sustained AF was highly reproducible, readily inducible and could be maintained for prolonged periods after CRAP. Coincidentally, Wijffels et al. [27] demonstrated that continuous RAP in the goat heart led to progressive shortening of the atrial effective refractory period and increased duration of AF once it was induced. The longer the duration of RAP, the longer the AF was maintained; this phenomenon was referred to as “AF begets AF”. Recently, the AF model produced by CRAP was used to study the mechanism of AF and to facilitate new therapeutic modalities [12], [28]. These studies suggested that atrial remodeling, including the significantly increased atrial fibrosis, inflammation, apoptosis, gap junctional remodeling and neural remodeling, has been produced by long-term RAP [12], [13], [14], [15]. We have previously demonstrated that RSD could suppress AF in canines with short-term RAP [26]. The present study also confirmed that RSD suppressed the AF vulnerability induced by CRAP. However, the role of renal denervation on atrial remodeling is unclear. Therefore, we discussed the long-term effect of RSD on atrial remodeling produced by prolonged RAP in canines.

Previous studies have demonstrated that catheter-based renal denervation could be a safe and effective means of substantially reducing blood pressure in patients with resistant hypertension [20], [21]. Our results showed that RSD had the direct effect of lowering SBP. RSD selectively reduces both renal sympathetic efferent and afferent nerve activity, which is indicated by a reduction in renal noradrenaline spillover measurements and plasma renin activity [29]. Thus, the beneficial effects of this procedure might extend beyond the reduction of blood pressure to other syndromes in which sympathetic hyperactivity is a component of the underlying disease process. Our data revealed that the plasma levels of Ang II and aldosterone were significantly reduced after RSD compared with the values for the CRAP group. Therefore, RSD can suppress the renin-angiotensin-aldosterone systematic activity.

It commonly means that higher concentration of Ang II in circulating plasma would increase the vessel contraction, increase the peripheral resistance to blood flow and eventually increase blood pressure. In the present study, the level of the Ang II in the endpoint of CRAP+RSD group and CRAP group were much higher than that in the endpoint of sham-operated group, however, the blood pressure in the CRAP+RSD group and CRAP group were lower than that in sham-operated group. In additon to the concentration of Ang II, there should be other mechanisms involved in the changes of blood pressure in CRAP+RSD group and CRAP group. Firstly, RSD reduces both renal sympathetic efferent and afferent nerve activity, and decreases the activity of RAAS. Secondly, RSD also affects sodium and water retention, resulting in declining blood volume [20], [21]. Last but not least, rapid right atrial pacing causes AF and cardiac dysfunction, further leading to hemodynamic changes. Atrial and ventricular ejection reduce and atria lost its normal effective contraction. Hence, lower BP in the CRAP+RSD group and CRAP group were observed in this study.

### Potential Mechanisms of RSD Effects on Atrial Remodeling

Recent findings have demonstrated a mechanistic link between inflammation and the development of AF, and AF is clearly associated with increased levels of known inflammatory markers, such as TNF-α and IL-6, even after adjustment for confounding factors. The RAAS plays a key role in this process. Furthermore, there is evidence that the angiotensin-converting enzyme inhibitors or angiotensin receptor blockers might be efficacious in the prevention of AF by modulating inflammatory pathways [15], [30]. The data in the present study showed that the left atrial levels of TNF-α and IL-6 were significantly increased in CRAP group compared with sham-operated group, but the increasing trend was inhibited in CRAP+RSD group compared with CRAP group. The anti-inflammatory effect of RSD treatment may have relationship with the suppressed plasma levels of Ang II and aldosterone. ANP is released in response to atrial stretch, and studies suggested that ANP has dose-dependent, autonomically mediated effects on atrial refractoriness and repolarization and plays a role in atrial fibrosis of AF [31], [32]. In addition, Our present study suggests that RSD treatment could attenuate the increase in left atrial level of ANP that is induced by CRAP and could markedly alleviate the lesion degree of cardic fibrosis compared with the CRAP group, thus suppressing atrial remodeling.

A previous study indicated that apoptotic death of myocytes contributes to cellular remodeling in fibrillating and dilated atria [33]. Both caspases and BCL-2 family proteins are involved in the apoptosis of cardiac myocytes, which occurs in various cardiopathies. Caspase-3 is one of the major caspases involved in apoptosis, and the biological process is regulated by the Bcl-2 family proteins: Bcl-2 can block cell death, while bax promotes apoptosis. Mano et al. [34]. demonstrated that the specific plasma membrane receptor for aldosterone is present on cardiac myocytes, and aldosterone accelerates the mitochondrial apoptotic pathway by activating calcineurin and the dephosphorylation of the calcineurin-Bad pathway. However, aldosterone-induced apoptosis can be prevented by blocking the effects of aldosterone and Ang II [35], [36]. Our results of apoptosis-associated protein expression and Tunel staining studies suggested that RSD treatment markedly reduced cardiocyte apoptosis produced by CRAP. This effect may be associated with the decreasing level of aldosterone and Ang II resulting from the inhibition of RAAS.

Previous work confirms that alterations of intercellular communication through gap junctional connections are likely contributing factors to the occurrence of AF. Moreover, Cx40 and Cx43 is abundant in atrium and make its contributions to atrial gap junctional remodeling [37]. Studies suggested that changes in expression of connexins accompany the chronification of AF, and the changes were partially or completely reversed after cardioversion of AF [38], [39]. The present study showed that RSD could reverse the upregulation of Cx40 expression induced by CRAP so as to suppress the gap junctional remodeling produced by prolonged RAP. A possible mechanism of this effect may be associated with the decreasing level of TNF-α resulting from the inhibition of RAAS, because TNF-α has been reported to close gap junction channels [40].

Series of studies [14], [41] suggest that neural remodeling contributes to the generation and maintence of AF. LS Chen et al. [14], [42] demonstrated that significant nerve sprouting and sympathetic hyperinnervation were present in a canine model of sustained AF produced by prolonged RAP. Immunohistochemistrical results in the present study showed that nerve sprouting and sympathetic hyperinnervation induced by prolonged RAP could be inhibited by RSD, suggesting its effects on the induction of cardiac denervation. One possible mechanism of cardiac denervation after RSD is the electrophysiological changes, which is related to the inhibition of RAAS [2].

### Clinical Implications

AF is recognized as the most common clinically significant cardiac arrhythmia. Current data estimated that the number of AF patients would continue to increase in the future, because the prevalence of AF increases with age and because of the aging population. So far, no ideal therapy can be applied to all patients with AF. Available antiarrhythmic drugs for preventing AF recurrence is far from ideal because of limited efficacy and potential side effects, particularly proarrhythmia [43]. Catheter ablation of AF is now a realistic therapeutic option in a broad spectrum of patients. Though the efficacy of pulmonary vein (PV) isolation and ganglionated plexi (GP) ablation may be more encouraging, they are at risks for major complications including potential recurrence, a small but nontrivial risk of pulmonary vein stenosis, systemic thromboembolism, pericardial effusion, cardiac tamponade, esophageal perforation, and phrenic nerve paralysis [44], [45]. These limitations stimulate research toward the development of effective but less aggressive procedures. In the study, we demonstrated that AF vulnerability was attenuated by RSD in rapid pacing-induced AF. Renal denervation may alter the atrial substrate remodeling by inhibiting RAAS activity. And previous studies have demonstrated that catheter-based RSD could be safe and effective [20], [21]. These present findings further support the assertion that ablation of extracardiac nerves is an effective alternative to the ablation of intracardiac nerves for the treatment of AF [26].

### Conclusions

In the study, we demonstrated that catheter-based renal denervation could suppress the renin-angiotensin-aldosterone systematic activity and atrial remodeling, including the significantly increased atrial fibrosis, inflammation and apoptosis, together with atrial gap junctional remodeling and neural remodeling that are produced by long-term RAP in ambulatory canines.

## References

[1] Schmieder RE, Hilgers KF, Schlaich MP, Schmidt BM (2007) Renin-angiotensin system and cardiovascular risk. Lancet 369: 1208–19. 17416265 10.1016/S0140-6736(07)60242-6

[2] Iravanian S, Dudley SC (2008) The Renin-Angiotensin-Aldosterone System (RAAS) and Cardiac Arrhythmias. Heart Rhythm 5: s12–s17. 18456194 10.1016/j.hrthm.2008.02.025 PMC2600881

[3] Boldt A, Wetzel U, Weigl J, Garbade J, Lauschke J, et al (2003) Expression of Angiotensin II Receptors in Human Left and Right Atrial Tissue in Atrial Fibrillation With and Without Underlying Mitral Valve Disease. J Am Coll Cardiol 42: 1785–92. 14642689 10.1016/j.jacc.2003.07.014

[4] Healey JS, Baranchuk A, Crystal E, Morillo CA, Garfinkle M, et al (2005) Prevention of Atrial Fibrillation With Angiotensin-Converting Enzyme Inhibitors and Angiotensin Receptor Blockers: A Meta-Analysis. J Am Coll Cardiol 45: 1832–39. 15936615 10.1016/j.jacc.2004.11.070

[5] Madrid AH, Bueno MG, Rebollo JM, Marin I, Pena G, et al (2002) Use of Irbesartan to Maintain Sinus Rhythm in Patients With Long-Lasting Persistent Atrial Fibrillation: A Prospective and Randomized Study. Circulation 106: 331–6. 12119249 10.1161/01.cir.0000022665.18619.83

[6] Belluzzi F, Sernesi L, Preti P, Salinaro F, Fonte ML, et al (2009) Prevention of Recurrent Lone Atrial Fibrillation by the Angiotensin-II Converting Enzyme Inhibitor Ramipril in Normotensive Patients. J Am Coll Cardiol 53: 24–29. 19118720 10.1016/j.jacc.2008.08.071

[7] Schneider MP, Hua TA, Bohm M, Wachtell K, Kjeldsen SE, et al (2010) Prevention of Atrial Fibrillation by Renin-Angiotensin System Inhibition: A Meta-Analysis. J Am Coll Cardiol 55: 2299–2307. 20488299 10.1016/j.jacc.2010.01.043

[8] Patlolla V, Alsheikhali AA, Alahmad AM (2006) The Renin-Angiotensin System: A Therapeutic Target in Atrial Fibrillation. PACE 29: 1006–12. 16981926 10.1111/j.1540-8159.2006.00477.x

[9] Nakashima H, Kumagai K, Urata H, Gondo N, Ideishi M, et al (2000) Angiotensin II Antagonist Prevents Electrical Remodeling in Atrial Fibrillation. Circulation 101: 2612–17. 10840013 10.1161/01.cir.101.22.2612

[10] Fukuda Y, Fukuda N, Morishita S, Tamura Y (2011) Preventive effect of renin–angiotensin system inhibitor on left atrial remodelling in patients with chronic atrial fibrillation: long-term echocardiographic study. European Journal of Echocardiography 12: 278–282. 21266378 10.1093/ejechocard/jeq193

[11] Morillo CA, Klein GJ, Jones DL, Guiraudon CM (1995) Chronic Rapid Atrial Pacing: Structural, Functional, and Electrophysiological Characteristics of a New Model of Sustained Atrial Fibrillation. Circulation 91: 1588–95. 7867201 10.1161/01.cir.91.5.1588

[12] Zhao J, Li J, Li W, Li Y, Shan H, et al (2010) Effects of spironolactone on atrial structural remodelling in a canine model of atrial fibrillation produced by prolonged atrial pacing. British Journal of Pharmacology 159: 1584–94. 20082611 10.1111/j.1476-5381.2009.00551.x PMC2925482

[13] Van der Velden HM, Ausma J, Rook MB, Hellemons AJ, Van veen TA, et al (2000) Gap junctional remodeling in relation to stabilization of atrial fibrillation in the goat. Cardiovasc Res 46: 476–486. 10912458 10.1016/s0008-6363(00)00026-2

[14] Chang C-M, Wu T-J, Zhou S-M, Doshi RN, Lee M-H, et al (2001) Nerve sprouting and symathetic hyperinnervation in a canine model of atrial fibrillation produced by prolonged right atrial pacing. Circulation 103: 22–25. 11136680 10.1161/01.cir.103.1.22

[15] Engelmann MD, Svendsen JH (2005) Inflammation in the genesis and perpetuation of atrial fibrillation. Eur Heart J 26: 2083–92. 15975993 10.1093/eurheartj/ehi350

[16] Kiryu M, Niwano S, Niwano H, Kishihara J, Aoyama Y, et al (2012) Angiotensin II-mediated up-regulation of connective tissue growth factor promotes atrial tissue fibrosis in the canine atrial fibrillation model. Europace 14: 1206–14. 22454409 10.1093/europace/eus052 PMC3404558

[17] Kasal DAB, Schiffrin EL (2012) Angiotensin II, Aldosterone, and Anti-Inflammatory Lymphocytes: Interplay and Therapeutic Opportunities. Int J Hypertens 2012: 132598–612. 10.1155/2012/829786 PMC3364573 22685633

[18] Velez Rueda JO, Palomeque J, Mattiazzi A (2012) Early apoptosis in different models of cardiac hypertrophy induced by high renin-angiotensin system activity involves CaMKII. J Appl Physiol 112 (12): 2110–20. 10.1152/japplphysiol.01383.2011 PMC3774203 22492934

[19] Sarkissian SD, Marchand EL, Duguay D, Hamet P, deBlois D (2003) Reversal of interstitial fibroblast hyperplasia via apoptosis in hypertensive rat heart with valsartan or enalapril. Cardiovas Res 57: 775–83. 10.1016/s0008-6363(02)00789-7 12618239

[20] Krum H, Schlaich M, Whitbourn R, Sobotka PA, Sadowski J, et al (2009) Catheter-based renal sympathetic denervation for resistant hypertension: a multicentre safety and proof-of-principle cohort study. Lancet 373: 1275–81. 19332353 10.1016/S0140-6736(09)60566-3

[21] Esler M, Krum H, Sobotka PA, Schlaich MP, Schmieder RE, et al (2010) Renal sympathetic denervation in patients with treatment-resistant hypertension (The Symplicity HTN-2 Trial): a randomised controlled trial. Lancet 373: 1903–9. 10.1016/S0140-6736(10)62039-9 21093036

[22] Davies JE, Manisty CH, Petraco R, Barron AJ, Unsworth B, et al (2013) First-in-man safety evaluation of renal denervation for chronic systolic heart failure: Primary outcome from REACH-Pilot study. Int J Cardiol 162(3): 189–92. 23031283 10.1016/j.ijcard.2012.09.019

[23] Brandt MC, Mahfoud F, Reda S, Schirmer SH, Erdmann E, et al (2011) Renal Sympathetic Denervation Reduces Left Ventricular Hypertrophy and Improves Cardiac Function in Patients With Resistant Hypertension. J Am Coll Cardiol 59: 901–9. 10.1016/j.jacc.2011.11.034 22381425

[24] Pokushalov E, Romanov A, Corbucci G, Artyomenko S, Baranova V, et al (2012) A randomized comparison of pulmonary vein isolation with versus without concomitant renal artery denervation in patients with refractory symptomatic atrial fibrillation and resistant hypertension. J Am Coll Cardiol 60: 1163–70. 22958958 10.1016/j.jacc.2012.05.036

[25] Hu J, Ji M, Niu C, Aini A, Zhou Q, et al (2012) Effects of Renal Sympathetic Denervation on Post-Myocardial Infarction Cardiac Remodeling in Rats. PLOS ONE 7(9): e45986–91. 23049914 10.1371/journal.pone.0045986 PMC3458818

[26] Zhao Q, Yu S, Zou M, Dai Z, Wang X, et al (2012) Effect of renal sympathetic denervation on the inducibility of atrial fibrillation during rapid atrial pacing. J Interv Card Electrophysiol 35(2): 119–125. 22869391 10.1007/s10840-012-9717-y

[27] Wijffels MC, Kirchhof CJ, Dorland R, Allessie MA (1995) Atrial fibrillation begets atrial fibrillation: a study in awake chronically instrumented goats. Circulation 92: 1954–68. 7671380 10.1161/01.cir.92.7.1954

[28] Zhang Y, Yamada H, Bibevski S, Zhuang S, Mowrey K, et al (2005) Chronic Atrioventricular Nodal Vagal Stimulation First Evidence for Long-Term Ventricular Rate Control in Canine Atrial Fibrillation Model. Circulation 112: 2904–11. 16260638 10.1161/CIRCULATIONAHA.105.568832

[29] Schlaich MP, Sobotka PA, Krum H, Lambert E, Esler MD (2009) Renal sympathetic-nerve ablation for uncontrolled hypertension. N Engl J Med 361: 932–934. 19710497 10.1056/NEJMc0904179

[30] Boos CJ, Anderson RA, Lip GY (2006) Is atrial fibrillation an inflammatory disorder? Eur Heart J 27(2): 136–149. 16278230 10.1093/eurheartj/ehi645

[31] Stambler BS, Guo GB (2005) Atrial Natriuretic Peptide Has Dose-Dependent, Autonomically Mediated Effects on Atrial Refractoriness and Repolarization in Anesthetized Dogs. J Cardiovasc Electrophysiol 16: 1341–47. 16403067 10.1111/j.1540-8167.2005.00259.x

[32] Cao H, Xue L, Wu Y, Ma H, Chen L, et al (2010) Natriuretic peptides and right atrial fibrosis in patients with paroxysmal versus persistent atrial fibrillation. Peptides 31: 1531–39. 20434499 10.1016/j.peptides.2010.04.019

[33] Aimé-Sempé C, Folliguet T, Rücker-Martin C, Krajewska M, Krajewskai S, et al (1999) Myocardial Cell Death in Fibrillating and Dilated Human Right Atria. J Am Coll Cardiol 34: 1577–86. 10551709 10.1016/s0735-1097(99)00382-4

[34] Mano A, Tatsumi T, Shiraishi J, Keira N, Nomura T, et al (2004) Aldosterone directly induces myocyte apoptosis through calcineurin-dependent pathways. Circulation 110: 317–323. 15249508 10.1161/01.CIR.0000135599.33787.CA

[35] Burniston JG, Saini A, Tan LB, Goldspink DF (2005) Aldosterone induces myocyte apoptosis in the heart and skeletal muscles of rats in vivo. J Mol Cell Cardiol 39: 395–399. 15907929 10.1016/j.yjmcc.2005.04.001

[36] Harada E, Yoshimura M, Yasue H, Nakagawa O, Nakagawa M, et al (2001) Aldosterone Induces Angiotensin-Converting-Enzyme Gene Expression in Cultured Neonatal Rat Cardiocytes. Circulation 104: 137–139. 11447075 10.1161/01.cir.104.2.137

[37] Duffy HS, Wit AL (2008) Is there a role for remodeled connexins in AF? No simple answers. J Mol Cell Cardiol 44: 4–13. 17935733 10.1016/j.yjmcc.2007.08.016 PMC2243184

[38] Polontchouk L, Haefliger JA, Ebelt B, Schaefer T, Stuhlmann D, et al (2001) Effects of Chronic Atrial Fibrillation on Gap Junction Distribution in Human and Rat Atria. J Am Coll Cardiol 38: 883–891. 11527649 10.1016/s0735-1097(01)01443-7

[39] Ausma J, van der Velden HM, Lenders MH, van Ankeren EP, Jongsma HJ, et al (2003) Reverse Structural and Gap-Junctional Remodeling After Prolonged Atrial Fibrillation in the Goat. Circulation 107: 2051–58. 12681996 10.1161/01.CIR.0000062689.04037.3F

[40] Chanson M, Berclaz PY, Scerri I, Dudez T, Wernke-Dollries K, et al (2001) Regulation of gap junctional communication by a pro-inflammatory cytokine in cystic fibrosis transmembrane conductance regulator-expressing but not cystic fibrosis airway cells. Am J Pathol 158 (5): 1775–84. 10.1016/S0002-9440(10)64133-8 PMC1891964 11337375

[41] Jayachandran JV, Sih HJ, Winkle W, Zipes DP, Hutchins GD, et al (2000) Atrial fibrillation produced by prolonged rapid atrial pacing is associated with heterogeneous changes in atrial sympathetic innervation. Circulation 101: 1185–91. 10715267 10.1161/01.cir.101.10.1185

[42] Tan AY, Zhou S, Ogawa M, Song J, Chu M, et al (2008) Neural Mechanisms of Paroxysmal Atrial Fibrillation and Paroxysmal Atrial Tachycardia in Ambulatory Canines. Circulation 118: 916–25. 18697820 10.1161/CIRCULATIONAHA.108.776203 PMC2742977

[43] Wyse DG, Waldo AL, DiMarco JP, Domanski MJ, Rosenberg Y, et al (2002) A comparison of rate control and rhythm control in patients with atrial fibrillation. N Engl J Med 347: 1825–33. 12466506 10.1056/NEJMoa021328

[44] Pappone C, Santinelli V, Manguso F, Vicedomini G, Guqliotta F, et al (2004) Pulmonary vein denervation enhances long-term benefit after circumferential ablation for paroxysmal atrial fibrillation. Circulation 109: 327–34. 14707026 10.1161/01.CIR.0000112641.16340.C7

[45] Katritsis DG, Giazitzoglou E, Zografos T, Pokushalov E, Po SS, et al (2011) Rapid pulmonary vein isolation combined with autonomic ganglia modification: a randomized study. Heart Rhythm 8(5): 672–8. 21199686 10.1016/j.hrthm.2010.12.047

